# Neutrophil-to-Lymphocyte Ratio Is an Independent Predictor of 30-Day Mortality of Intracerebral Hemorrhage Patients: a Validation Cohort Study

**DOI:** 10.1007/s12640-018-9890-6

**Published:** 2018-03-28

**Authors:** Fei Wang, Li Wang, Ting-ting Jiang, Jian-jun Xia, Feng Xu, Li-juan Shen, Wen-hui Kang, Yong Ding, Li-xia Mei, Xue-feng Ju, Shan-you Hu, Xiao Wu

**Affiliations:** 1 Department of Critical Care Medicine, Jiading District Central Hospital Affiliated Shanghai University of Medicine & Health Sciences, Shanghai, China; 2 Emergency Department, Jiading District Central Hospital Affiliated Shanghai University of Medicine & Health Sciences, Shanghai, China; 3 Department of Clinical Laboratory, Jiading District Central Hospital Affiliated Shanghai University of Medicine & Health Sciences, Shanghai, China; 4 Jiading Town Community Healthcare Center, Jiading District, Shanghai, China

**Keywords:** Neutrophil-to-lymphocyte ratio, Intracerebral hemorrhage, 30-day mortality, Inflammation

## Abstract

In a previous study in patients with intracranial hemorrhage (ICH), we found an association between high neutrophil-to-lymphocyte ratio (NLR) with poor short-term mortality. In the current study, this preliminary finding was validated using an independent patient cohort. A total of 181 ICH patients (from January 2016 to December 2017) were included. Diagnosis was confirmed using computed tomography (CT) in all cases. Patient survival (up to 30 days) was compared between subjects with high NLR (above the 7.35 cutoff; *n* = 74) versus low NLR (≤ 7.35; *n* = 107) using Kaplan-Meier analysis. A multivariate logistic regression was performed to identify factors that influenced the 30-day mortality. Correlation between NLR with other relevant factors (e.g., C-reactive protein (CRP) and fibrinogen) was examined using Spearman correlation analysis. The 30-day mortality was 19.3% (35/181) in the entire sample, 37.8% (28/74) in the high-NLR group, and 6.5% (7/107) in the low-NLR group (*P* < 0.001). In comparison to the low-NLR group, the high-NLR group had higher rate of intraventricular hemorrhage (29.7 vs. 16.8%), ICH volume (median 23.9 vs. 6.0 cm^3^) and ICH score (median 1.5 vs. 0), and lower GCS score (9.4 ± 4.5 vs. 12.9 ± 3.2). An analysis that divided the samples into three equal parts based on NLR also showed increasing 30-day mortality with incremental NLR (1.6, 15.0, and 41.7% from lowest to highest NLR tertile, *P* for trend < 0.001). Kaplan-Meier curve showed higher 30-day mortality in subjects with high NLR than those with low NLR (*P* < 0.001 vs. low-NLR group, log-rank test). High NLR (> 7.35) is associated with poor short-term survival in acute ICH patients.

## Introduction

Acute intracerebral hemorrhage (ICH) is associated with high disability and mortality. Increasing evidence suggests that inflammation contributes significantly to tissue damage caused by ICH. Specifically, activated inflammatory cells could release a variety of proinflammatory cytokines and proteases (Zhao et al. 2007), which in turn cause secondary brain injury. Edema, typically the result of inflammatory responses and mechanical compression by hematoma, is a major clinical feature of secondary brain injury and contributes to neurological deterioration (Babu et al. 2012).

Neutrophil-to-lymphocyte ratio (NLR) increases with increasing severity of inflammatory response and has been associated with poor patient outcomes in cancers (Grenader et al. 2016; Ojerholm et al. 2017), cardiovascular diseases (Kurtul et al. 2016; Sari et al. 2015), ischemic diseases (Aktimur et al. 2016; Qun et al. 2017), and a variety of other conditions (Ozcicek et al. 2017; Pan et al. 2017; Senturk et al. 2016). A recent study from this research group (Wang et al. 2016) showed an association of high NLR with 30-day mortality in ICH patients. High NLR has also been suggested to be predictive for 90-day prognosis (Lattanzi et al. 2016b and early neurological deterioration in patients with acute ICH (Lattanzi et al. 2017b). In a study by Lattanzi et al. (2018), NLR improved the accuracy of outcome prediction when added to the Modified ICH score. In patients with ischemic stroke, high NLR has also been associated with bleeding after thrombolysis (Guo et al. 2016). In the current study, we used an independent cohort of ICH patients to validate our previous finding that high NLR (> 7.35) is associated with 30-day mortality in ICH patients.

## Methods

### Study Sample

Consecutive adult ICH patients receiving treatment for acute ICH at the Emergency Department of Jiading District Central Hospital Affiliated Shanghai University of Medicine & Health Sciences between January 2016 and December 2017 were retrospectively reviewed. The diagnosis of ICH was established with CT scan in all subjects. The inclusion and exclusion criteria are listed in Table 1.

**Table 1:** Inclusion and exclusion criteria of the study participants

Inclusion criteria
Patients with a diagnosis of intracerebral hemorrhage (ICH) verified by CT scans.
Age ≥ 18 years
Exclusion criteria
Patients admitted to the hospital **>** 24 h after ICH.
Patients with hematologic disorders, immunosuppressant drug users (steroids), those with a history of infection within 2 weeks before ICH, a stroke history within 6 months, patients with a history of malignancy, and those using anticoagulants.
Patients who refused treatment.

The study protocol was approved by the Ethics Review Board of Jiading District Central Hospital (No.2017-KY-09). All subjects were de-anonymized. Written informed consent was waived by the Ethics Review Board.

### Data Collection

Demographic information, past medical history, clinical data, and laboratory measures were collected from medical records. Hypertension was defined using the 2013 ESH/ESC Guidelines (Mancia et al. 2014): resting systolic pressure (SBP) at ≥ 140 mmHg and/or diastolic pressure (DBP) at ≥ 90 mmHg on three separate occasions or regular use of anti-hypertension medications. Diabetes was defined using the 2016 American Diabetes Association Guidelines (Chamberlain et al. 2016). All laboratory tests were carried out using venous blood collected after over-night fasting. Patient management was, in principle, based on the 2015 American Heart Association/American Stroke Association Guidelines (Hemphill 3rd et al. 2015).

### Imaging Analysis

The ICH diagnosis was based on clinical features and confirmed by a post hoc assessment of CT images by an experienced neurologist. The following features were extracted using the CT slice with the largest ICH area: (A) the largest diameter of the hematoma; (B) the dimension of the hemorrhage perpendicular to the largest diameter as the second diameter; (C) the height of the hematoma, as calculated by multiplying the number of slices involved by the slice thickness. ICH volume was calculated as follows: ABC/2 (Kothari et al. 1996). Intraventricular hemorrhage (IVH) was defined as hyperdense intraventricular signal not attributable to calcification or choroid plexus.

### Statistical Analysis

Based on our previous study (Wang et al. 2016), the study sample was divided using NLR at a cutoff of 7.35. Continuous variables were analyzed using Student’s *t* test if normally distributed and with Mann-Whitney *U* test if otherwise. Categorical variables were analyzed using *χ*^2^ test. Potential association between NLR and 30-day mortality was also assessed by dividing the sample into three parts of equal size followed by *P* for trend analysis using the Jonckheere-Terpstra test. Spearman correlation analysis was used to determine the correlation of NLR with other factors. Multiple logistic regression was conducted to identify the factors that influenced the 30-day mortality. *P* < 0.05 was considered statistically significant. All statistical analyses were performed using IBM SPSS 19.0 (IBM, Armonk, New York, USA).

## Results

A total of 213 patients with acute ICH sought emergency care at our department during the study period; 32 patients were excluded due to treatment discontinuation within 24 h (*n* = 19), hospital admission at **>** 24 h after the first symptom (*n* = 2), infection within 2 weeks before ICH (*n* = 5), anticoagulant use within 3 months (*n* = 5), and leukemia (*n* = 1). The final analysis included 181 patients (112 men; age 65.8 ± 14.3 years). The mean duration from disease onset to sample collection was 14.8 ± 6.9 h (range: 4–22). The total 30-day mortality was 19.3% (35/181). Demographic data and clinical features are shown in Table 2.

**Table 2:** Characteristics of all ICH patients included in the study

Characteristics (*n* = 181)	
Age (years); mean ± SD; (range)	65.8 ± 14.3 (29~91)
Age ≥ 80 years [*n* (%)]	39 (21.5)
Male [*n* (%)]	112 (61.9)
Hypertension [*n* (%)]	156 (86.2)
Diabetes mellitus [*n* (%)]	43 (23.8)
30-day mortality [*n* (%)]	35 (19.3)
Supratentorial origin [*n* (%)]	166 (91.7)
Presence of IVH [*n* (%)]	40 (22.1)
ICH volume (cm^3^); mean ± SD; (range)	23.8 ± 35.2 (2.3~180.8)
GCS score, mean ± SD; (range)	11.5 ± 4.2 (3~15)
ICH score, mean ± SD; (range)	1.3 ± 1.4 (0~5)
Time from ICH onset to sample collect, hours	14.8 ± 6.9 (4~22)
Systolic BP (mmHg); mean ± SD; (range)	139 ± 15 (92~223)
Diastolic BP (mmHg); mean ± SD; (range)	81 ± 16 (57~112)
WBC (*10^9^/L); mean ± SD; (range)	9.6 ± 4.5 (4.6~48.1)
Neutrophil (*10^9^/L); mean ± SD; (range)	7.6 ± 4.5 (0.6~41.4)
Lymphocyte (*10^9^/L); mean ± SD; (range)	1.2 ± 0.5 (0.2~3.3)
NLR; mean ± SD; (range)	8.7 ± 8.6 (1.0~61.9)
CRP (mg/L); mean ± SD; (range)	32.7 ± 8.6 (1.0~198.0)
Fibrinogen (mg/dl); mean ± SD; (range)	3.6 ± 0.9 (1.4~6.9)

**Fig. 1:**
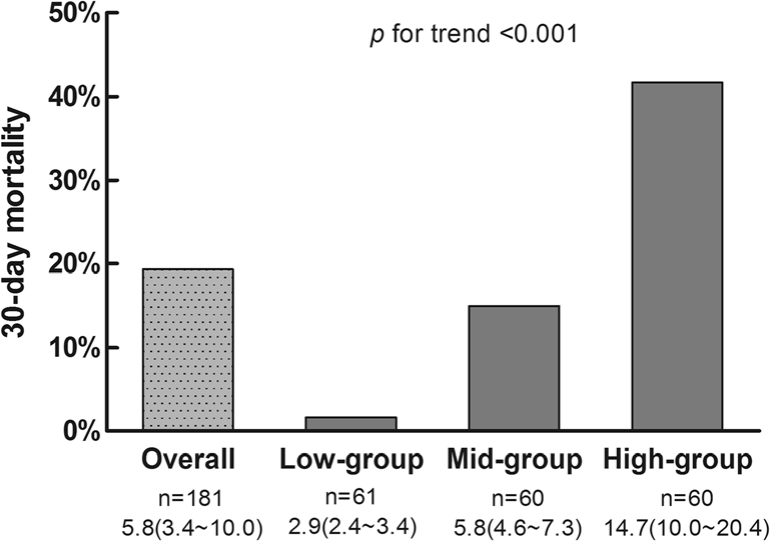
The trend for 30-day mortality with increasing NLR, from the lowest to highest tertile (*n* = 61 or 60 per tertile). The median value and the 25th~75th are shown under the label of horizon axis

The study sample was divided into three parts of equal size based on NLR: lowest (NLR median: 2.9, 25th~75th: 2.4~3.4), middle (NLR median: 5.8, 25th~75th: 4.6~7.3), and highest (NLR median: 14.7, 25th~75th: 10.0–20.4). The 30-day mortality was 1.6, 15, and 41.7% in the groups with lowest, middle, and highest NLR, respectively (Fig. 1). *P* for trend was < 0.001.

Among the 181 patients, 74 had high NLR (> 7.35); the remaining 107 had low NLR (≤ 7.35). CRP and fibrinogen data were only available in 136 (75%) and 119 (66%) cases out of the 181 total cases, respectively. The 30-day mortality was 37.8% (28/74) in the high-NLR group vs. 6.5% (7/107) in the low-NLR group (*P* < 0.001). The two groups also differed significantly in the rate of IVH (29.7 vs. 16.8%), ICH volume (median 23.9 vs. 6 cm^3^), ICH score (median 2 vs. 0), GCS score (9.4 ± 4.5 vs. 12.9 ± 3.2), WBC (median 11.8 × 10^9^/L vs. 8.3 × 10^9^/L), neutrophil count (median 9.7 × 10^9^/L vs. 5.1 × 10^9^/L), lymphocyte count (0.8 × 10^9^/L vs. 1.4 × 10^9^/L), CRP (29 vs. 6 mg/L) (Table 3).

**Table 3:** Clinical characteristics of population with NLR ≤ 7.35 and NLR > 7.35

	Low-NLR group (≤ 7.35, *n* = 107)	High-NLR group (> 7.35, *n* = 74)	*P*
Age (years); mean ± SD	65.0 ± 13.9	67.1 ± 14.8	0.327
Age ≥ 80 years [*n* (%)]	20 (18.7)	19 (25.7)	0.261
Male [*n* (%)]	65 (60.7)	47 (63.5)	0.706
Hypertension [*n* (%)]	93 (86.9)	63 (85.1)	0.733
Diabetes mellitus [*n* (%)]	25 (23.4)	18 (24.3)	0.881
Supratentorial origin [*n* (%)]	96 (89.7)	70 (94.6)	0.242
Presence of IVH [*n* (%)]	18 (16.8)	22 (29.7)	0.040
ICH volume (cm^3^); median (IQR)	6.0 (10.9)	23.9 (41.3)	< 0.001
GCS score, mean ± SD	12.9 ± 3.2	9.4 ± 4.5	< 0.001
ICH score, median (IQR)	0 (1)	1.5 (2)	< 0.001
Time from ICH onset to sample collection, hours, mean ± SD	14.0 ± 6.8	15.9 ± 7.0	0.066
Systolic BP (mmHg), mean ± SD	157.5 ± 24.2	161.5 ± 26.3	0.297
Diastolic BP (mmHg), mean ± SD	89.1 ± 14.6	95.9 ± 17.0	0.005
WBC (*10^9^/L), median (IQR)	8.3 (4.2)	11.8 (6.5)	< 0.001
Neutrophil (*10^9^/L), median (IQR)	5.1 (2.9)	9.7 (5.1)	< 0.001
Lymphocyte (*10^9^/L), median (IQR)	1.4 (0.5)	0.8 (0.4)	< 0.001
NLR, median (IQR)	3.7 (2.2)	11.5 (11.1)	< 0.001
CRP (mg/L); median (IQR)	6 (16.5)	29 (68.5)	< 0.001
Fibrinogen (mg/dl); mean ± SD	3.4 ± 0.9	3.7 ± 1.0	0.114
30-day mortality [*n* (%)]	7 (6.5)	28 (37.8)	< 0.001

The Spearman correlation analysis showed an association between NLR with the presence of IVH, ICH volume, GCS score, ICH score, and 30-day mortality as well as CRP (Table 4).

**Table 4:** The correlation between NLR, NLR > 7.35, and other factors

Factors	NLR		NLR > 7.35	
	Spearman’s rho	*P*	Spearman’s rho	*P*
Presence of IVH	0.297	< 0.001	0.166	0.026
ICH volume	0.572	< 0.001	0.474	< 0.001
GCS score	− 0.533	< 0.001	− 0.417	< 0.001
ICH score	0.444	< 0.001	0.347	< 0.001
30-day mortality	0.454	< 0.001	0.390	< 0.001
CRP	0.566	< 0.001	0.487	< 0.001
Fibrinogen	0.090	0.376	0.161	0.114

We conducted a logistic regression analysis that included NLR (high vs. low), age (≥ 80 years vs. below), IVH (presence vs. absence), ICH volume (≥ 30 cm^3^ vs. below), GCS score, SBP, DBP, and WBC as independent variables. Selection of the factors was based previously reported association with clinical outcome in ICH patients (Wang et al. 2016; Lattanzi et al. 2016a, b). After adjustment for other factors, high NLR remained to be associated with 30-day mortality, with an odds ratio (OR) of 3.797 (95% CI 1.280–11.260) (Table 5). Other factors associated with high mortality included the following: ICH volume ≥ 30 cm^3^ (OR 2.979, 95% CI 1.012–8.767) and GCS score (OR 0.862, 95% CI 0.755–0.984).

**Table 5:** Adjusted risk factors for 30-day mortality in ICH patients

Variables	*P*	OR	95% CI
Presence of IVH	0.003	7.249	1.983–26.504
ICH volume ≥ 30 cm^3^	0.021	15.381	1.502–157.493
GCS score	0.003	0.713	0.570–0.893
NLR > 7.35	0.011	8.365	1.623–43.110
CRP	0.081	1.014	0.998–1.029

**Fig. 2:**
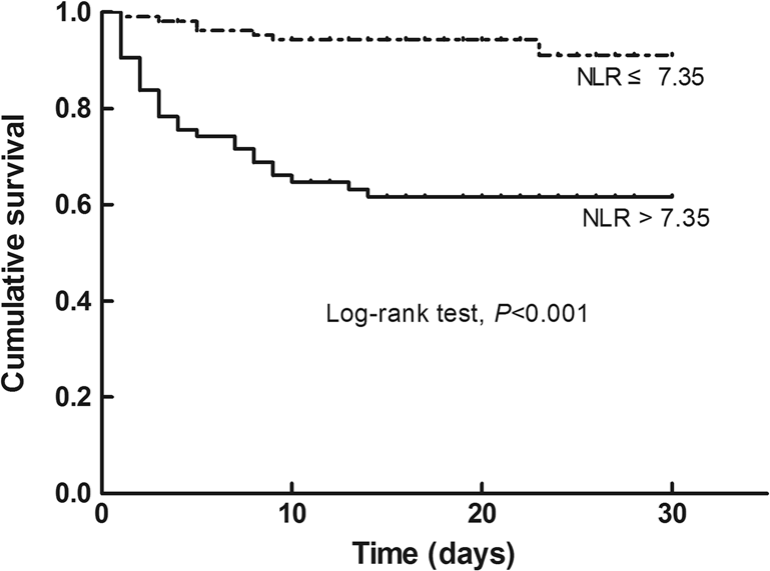
Kaplan-Meier curve showing 30-day mortality in subjects with low NLR (≤ 7.35; dotted line; *n* = 107) vs. high NLR (> 7.35; solid line; *n* = 74)

The Kaplan-Meier analysis showed that patients with high NLR had significantly higher 30-day mortality than those with low NLR (log-rank test, *P* < 0.001, Fig. 2).

## Discussion

Previous studies indicated that NLR is closely related to the prognosis of stroke patients (Aktimur et al. 2016; Qun et al. 2017). High NLR is associated with 30-day mortality (Wang et al. 2016) and in-hospital mortality (Giede-Jeppe et al. 2017), as well as 90-day mortality (Lattanzi et al. 2016a, b; Tao et al. 2017) in ICH patients. In patients with ischemic stroke, high NLR has also been associated with hemorrhagic transformation upon thrombolysis (Guo et al. 2016). In the current study, we found a close association of high NLR (> 7.35) with IVH, ICH volume, and ICH score. We also identified a negative correlation between NLR and GCS score. Multivariate logistic regression showed that high NLR is an independent risk for 30-day mortality.

The association between high NLR and short-term mortality is highly complex and could involve many other factors. Upon ICH, neutrophils are the earliest WBCs that appear in hematoma (Wang 2010), peaking in 2–3 days and then gradually disappearing (Wang and Dore 2007; Zhou et al. 2014). Neutrophils release large amounts of tumor necrosis factor-α (TNF-α). The concentration of TNF-α in plasma is positively correlated with ICH volume (Behrouz 2016). There is also a positive correlation between the number of TNF-α positive cells and apoptotic neurons around the hematoma (Zhang et al. 2015).

Neutrophils could aggravate brain damage by producing reactive oxygen species, releasing proinflammatory factors, upregulating the expression of metalloproteinase 9, and increasing blood-brain barrier permeability (Moxon-Emre and Schlichter 2011). Neutrophils could also stimulate microglia/macrophages to release a variety of cytokines and free radicals (Wang and Dore 2007). High interleukin-1β (IL-1β) could exacerbate brain edema through inflammatory response and increasing blood-brain barrier permeability (Wei et al. 2014). In a study in animal model of ICH, lymphocytes potentiated cerebral inflammation and brain injury (Rolland 2nd et al. 2011). Fingolimod (Thomas et al. 2017), a drug that reduces T cell cycle pool, could reduce brain edema by downregulating inflammatory mediators, including γ-interferon, IL-17, and expression of intracellular adhesion molecules (Rolland et al. 2013).

Decreased lymphocyte count has been reported to be associated with 90-day mortality (Morotti et al. 2017b) and poor neurological recovery (Giede-Jeppe et al. 2016) in ICH patients. Lower lymphocyte count in non-survivors identified in the current study is consistent with these previous reports. As an established easy-to-use marker of systemic inflammation (Celikbilek et al. 2014), NLR conveys important information about the complex inflammatory activity in the vascular bed (Tamhane et al. 2008).

The current study had several limitations. First, it is an observational, single-institution study with relatively small sample size. Second, we did not examine the relationship between NLR and proinflammatory cytokines. Third, a multitude of variables acts at both local and systemic level to interfere with the pathways linked to the secondary damage and neurovascular recovery (Lattanzi et al. 2013; Lattanzi et al. 2016a; Zangari et al. 2016). Many of these variables were not analyzed in the current study. For example, hematoma growth after ICH has been associated with neuroimaging features (e.g., spot sign (Ciura et al. 2014) and several non-contrast CT markers (Morotti et al. 2017a) as well as blood pressure management (Lattanzi et al. 2017a). Blood pressure variability has been associated with poor clinical outcome both in patients with ischemic stroke (Buratti et al. 2014) and ICH (Lattanzi and Silvestrini 2015; Lattanzi and Silvestrini 2016; Lattanzi et al. 2015). Unfortunately, the current study is based on routine clinical practice in which blood pressure was not measured continuously.

In summary, we found higher 30-day mortality in ICH patients with high NLR (> 7.35). Multivariate regression showed that high NLR is an independent risk for 30-day mortality.

## Abbreviations

ICH: intracerebral hemorrhage
NLR: neutrophil-to-lymphocyte ratio
GCS: Glasgow Coma Scale
IVH: intraventricular hemorrhage
CT: computed tomography
SBP: systolic pressure
DBP: diastolic pressure
OR: odds ratios
CI: confidence intervals
WBC: white blood cells
CRP: C-reactive protein
TNF-α: tumor necrosis factor-α
IL-1β: interleukin-1β
